# Using flash glucose monitoring in pregnancies in routine care of patients with gestational diabetes mellitus: a pilot study

**DOI:** 10.1007/s00592-023-02042-x

**Published:** 2023-02-25

**Authors:** Sophie Bastobbe, Yvonne Heimann, Ekkehard Schleußner, Tanja Groten, Friederike Weschenfelder

**Affiliations:** Department of Obstetrics, University Hospital Jena, Am Klinikum 1, 07747 Jena, Germany

**Keywords:** Gestational diabetes, Flash glucose monitoring, FGM, SMBG, Treatment, Perinatal outcome, Treatment satisfaction

## Abstract

**Aim:**

Flash glucose monitoring (FGM) has been approved for the care of pregnant women with preexisting diabetes since 2017. However, its use in gestational diabetes (GDM) has been critically discussed. Inaccuracy and missing recommendations for target values are the main arguments against the use of FGM in GDM. To date, there is a lack of data to justify routine use of FGM in GDM pregnancies. Consequently, this new technology has been withheld from GDM-patients. Aim of our pilot study was to analyze the impact of FGM use on pregnancy outcomes, patient’s satisfaction and to confirm the safe use in GDM pregnancies.

**Methods:**

Cohort study of 37 FGM-managed GDM pregnancies compared with 74 matched women using self-monitoring of blood glucose (SMBG). Group comparison using nonparametric testing concerning patients characteristic and perinatal outcome focusing on adverse outcomes (preeclampsia, preterm delivery, large for gestational age, C-sections, neonatal intensive care unit admission, hyperbilirubinemia and hypoglycemia). Evaluation of patient’s treatment satisfaction using the “Diabetes Treatment Satisfaction Questionnaire change” (DTSQc) and patient interviews.

**Results:**

No significant differences in patient’s characteristics despite gestational age at diagnosis (FGM with 20 vs. SMBG with 25 weeks). No difference in gestational weight gain, HbA1c progression and perinatal outcome. Treatment satisfaction obtained by the DTSQc revealed a high level of satisfaction with FGM use.

**Conclusion:**

FGM use was well accepted and did not affect perinatal outcome. Use of FGM during pregnancy is safe and non-inferior to the management with SBGM. FGM should be considered as an option in the management of GDM patients.

## Introduction

In recent years, a significant increase in the incidence of gestational diabetes mellitus (GDM) has been recorded. Depending on different definitions and diagnostic criteria, the prevalence is determined to be 7–10% in pregnancies and even exceeding 10% when the diagnostic threshold used in the IADPSG criteria is applied [1, 2]. GDM is triggered by an inability to adapt to the hormonal and physiological changes caused by pregnancy leading to a relative insulin deficiency and subsequent hyperglycemia. Favoring factors for this inability to adapt may be the increasing incidence of obesity, the general decline in physical activity, and the trend toward later motherhood [3, 4]. The resulting high maternal blood sugar levels lead to an excess of glucose for the child, which negatively affects fetal development and significantly increases the risk of complications during delivery and the neonatal period. Due to maternal hyperglycemia and resulting anabolic effects supported by greatly increased fetal insulin levels, there is an increased risk for fetal maladaptation and large for gestational age babies (LGA, birth weight > 90th percentile) [5]. LGA births are associated with an increased risk of birth trauma and cesarean section, but also with a greater proportion of shoulder dystocia [6]. In addition, perinatal complications such as respiratory distress, hypoglycemia, or jaundice are more common in these newborns [7, 8]. Long-term consequences for children of diabetic pregnancies such as persistent impairment of glucose tolerance [9] and a reduction in intellectual performance have also been demonstrated [10]. Sufficient glycemic control in the mother during pregnancy however, has been shown to significantly reduce the impact of diabetes on short-term [11–13] and long-term outcome [14–17]. To achieve the goal to avoid maternal hyperglycemia in pregnancy according to the German S3 guidelines, lifestyle modifications with increased physical activity, dietary changes, regular glucose monitoring, follow-up appointments, and, if necessary, insulin therapy [18] are the key pillars of GDM management. These recommendations represent a significant intervention into the daily life of affected women and can be perceived as time-consuming and disruptive, not least because of the need for the painful self-measurement of blood glucose levels by finger pricking (SMBG), often performed in presence of other people. Patients reported an increased expenditure of time, pain, an intrusion into everyday life, a feeling of stigmatization, restriction in autonomy due to constant monitoring of the values and, last but not least, the psychological burden due to fears arising for their own and their child’s health [19]. However, regular monitoring of glucose levels is essential for successful treatment, since GDM therapy is based on these values recorded by patients. In addition, self-measured values represent a biofeedback by visualizing the effect of lifestyle interventions as well as physiological metabolic changes during the course of pregnancy. Accordingly studies have shown that lower glucose levels correlate positively with patient satisfaction and mental health, proving that optimization of therapy has an impact on maternal metabolism and consequently on perinatal and neonatal outcome [20]. However, the use of SMBG biofeedback is always time delayed, whereas flash glucose monitoring (FGM) biofeedback is instantaneous and offers the possibility of direct observation.

With the establishment of FGM it is possible for diabetics to significantly reduce finger pricking. Sensors are inserted into the subcutaneous fat tissue for up to 14 days, which continuously generates glucose values that can be transmitted to the respective reader by “scanning” [21]. Since 2017, FGM has been approved for pregnant diabetic women and has also been shown to be a successful therapy in studies [22, 23]. However, using FGM in the care of GDM—patients is critically debated and currently still decided on an individual basis [24]. FGM—devices could allow GDM patients to better integrate management recommendations into daily life and relieve the burden of SBGM. Furthermore, through biofeedback, generated by directly monitoring of glucose response to physical activity or food intake, GDM patients get the opportunity to develop self-directed management strategies to help them achieve target levels. Additionally, the increase in information on glucose values during the entire day will help intervene more specifically meeting the individual demands of patients. To date, there is a lack of systematically collected data on the use of FGM, maternal and infant outcomes, and patient satisfaction that summarizes all parameters so that routine use could be warranted.

The aim of our pilot study is to demonstrate the feasibility and equivalence of perinatal outcomes of FGM use compared with SMBG by presenting a cohort of 37 GDM pregnancies monitored with an FGM device. In addition, we wanted to assess the subjective evaluation and satisfaction of the patients in order to evaluate whether the assumed facilitation in management due to FGM use is perceived in our cohort.

## Research design and methods

### Study population

Between 2019 and 2021, 37 patients cared for GDM at our competence center for diabetes and pregnancy received FGM device for monitoring glucose levels during their pregnancy. GDM diagnosis was based on the IADPSG and WHO 2013 criteria [25]. Initially all GDM patients were trained to perform SMBG. Women needing insulin or had a high risk for insulin dependency due to a history of GDM, an early GDM diagnosis or risk factors for type 2 diabetes were randomly offered FGM use. All FGM patients received individual training on FGM and were advised to continue measuring fasting glucose by SMBG to ensure that FGM readings are reliable as well as in situations with implausible glucose values [26]. Diabetes care was provided according to the 2011 German S3 guideline and was performed by our hospital outpatient clinic [27]. Patients without FGM during that time period (*n* = 288) performed SMBG four times a day or seven times in case of insulin treatment. Patients were monitored on a four-week basis in case of diet control and fortnightly if insulin treatment became necessary. Of the 288 SMBG users 74 were finally matched 1:2 according to maternal age, body mass index (BMI), HbA1c at diagnosis and treatment. Ethical approval was obtained from the Ethics Committee of Friedrich Schiller University in Jena, Germany (Reg.-Nr. 2022-2826 12/22).

### Study data collection

Patient’s characteristics were retrieved from patient records. BMI was calculated using pre-pregnancy weight and height of the patients and grouped according to the definitions of the world health organization [28]. Gestational weight gain (GWG) was calculated from the difference between the pre-pregnancy weight and the last documented weight before delivery. Relevant maternal characteristics were the following parameters: age, BMI, obesity, GWG, excessive weight gain according to IOM recommendations and proportions of insulin-dependent patients. HbA1c levels according to IFCC or NGSP/DCCT standard were determined on a four weekly basis in a standardized setting and are presents as follows: HbA1c at diagnosis (%), HbA1c at delivery (%) and HbA1c changes, calculated as a difference between the two before mentioned measurements.

The following endpoints were defined for perinatal complications: hypertensive disorders of pregnancy (HDP; including preeclampsia, pregnancy induced hypertension and HELLP syndrome), preterm delivery (defined as delivery before 37 weeks of gestation) and C-section. Fetal outcome included APGAR 5, hyperbilirubinemia, hypoglycemia, admission to neonatale intensive care unit (NICU) and a neonatal weight above the 90th percentile, defining large for gestational age (LGA), using Voigt’s percentiles [29]. Patient’s satisfaction with individual GDM monitoring was evaluated using a modified version of the “Diabetes Treatment Satisfaction Questionnaire change” (DTSQc).

### Study questionnaire

Treatment satisfaction was retrospectively obtained using an adapted version of the DTSQc after pregnancy. DTSQc collects patients answers on their satisfaction with their new treatment (FGM) compared with their previous treatment (SMBG). Patients could choose on a numerical scale from 3 to − 3 (meaning “much more satisfied now” to “much less satisfied now”). In the second section of our questionnaire, GDM patients could indicate in free text fields what they liked less or especially about the sensor-based measurement.

### Statistical analysis

Statistical analysis was performed with SPSS 27.0 (IBM Corp. Released 2016. IBM SPSS Statistics for Windows, Version 24.0. Armonk, NY: IBM Corp). Chi^2^ test or Fisher exact test was used to compare categorical data. Since most of the data were not normally distributed median and interquartile range was used for data presentation and description. To compare subgroups nonparametric tests were performed to compare continuous data. A *p* value < 0.05 was considered to indicate statistical significance (2-tailed).

## Results

### Patient characteristics and perinatal outcomes

Patient and pregnancy characteristics, as well as perinatal outcome are summarized in Table 1. Analysis of the two subgroups for patient characteristics revealed no significant differences in age, pre-pregnancy BMI, previous births, GWG and excessive gestational weight gain. (Table 1) Diagnosis of GDM occurred significantly earlier in women in the FGM- subgroup (median 20 (IQR 14–26) vs. median 25 (IQR 18–28); *p* = 0.044). HbA1c levels at birth were higher in FGM monitored pregnancies [5.6% (IQR 5.4–5.8) vs. 5.4% (IQR 5.2–5.6) or 38 mmol/mol vs. 36 mmol/mol; *p* = 0.013], whereas HbA1c- values at diagnosis and change in HbA1c did not significantly differ between FGM and SMBG groups (HbA1c at diagnosis 5.4% (36 mmol/mol) vs. 5.3% (34 mmol/mol) and change in value: 0.3 vs. 0.1). Perinatal outcome parameters did not differ between the two subgroups.

**Table 1:** Baseline characteristics, pregnancy and perinatal outcome of the subgroups FGM (*n* = 74) and SMBG (*n* = 74) subgroup

Variables	Available data	FGM (*n* = 37)	SMBG (*n* = 74)	*p*
Age in years		34 (32–37)	35 (31–37)	0.331
Pre-pregnancy BMI in kg/m^2^		31 (28–40)	31 (27–36)	0.695
Obesity		21 (56.8%)	40 (54.1%)	0.963
Parity		1 (1–2)	1 (0–1)	0.320
GWG in kg		10.6 (5.2–15)	12 (7–17)	0.573
GA at diagnosis		20 (14–26)	25 (18–28)	0.044*
Excessive GWG	(93/30/63)	40% (12)	49% (31)	0.506
Need for Insulin		86.5% (32)	86.5% (64)	1
HbA1c at diagnosis %		5.4 (5.1–5.5)	5.3 (5.1–5.4)	0.094
HbA1c at diagnosis mmol/mol		36 (32–37)	34 (32–36)	
HbA1c at delivery in %		5.6 (5.4–5.8)	5.4 (5.2–5.6)	0.013*
HbA1c at delivery in mmol/mol		38 (36–40)	36 (33–38)	
HbA1c changes in %		0.3 (0.1–0.4)	0.1 (0–0.4)	0.109
Hypertensive Pregnancy Disorders	(105/32/73)	6.3%(2)	5.5%(4)	1
Preterm delivery	(102/30/72)	3.3% (1)	2.8% (2)	1
C-Section	(103/73/30)	26.7% (8)	42.5% (31)	0.180
GA at delivery		38 (37–39)	39 (38–40)	0.430
Birth weight in g		3510 (3249–3838)	3523 (3115–3929)	0.971
LGA	(103/73/30)	20% (6)	13.7% (10)	0.550
APGAR 5		10 (9–10)	9 (9–10)	0.078
hyperbilirubinemia	(89/28/61)	35.7% (10)	44.3% (27)	0.494
hypoglycemia	(94/29/65)	2 (6.9%)	1 (1.5%)	0.224
NICU admission	(103/30/73)	1 (3.3%)	13.7% (10)	0.169

Data are *n* (%) or median (interquartile range) unless otherwise specified. p* Comparison of FGM vs. SMBG; *p* < 0.05 is significant and bold

*FGM* flash glucose monitoring, *GA* gestational age, *GDM* gestational diabetes, *GWG* gestational weight gain, *LGA* large for gestational age, *NICU* neonatal intensive care unit, and *SMBG* self-monitoring of blood glucose

### Diabetes treatment satisfaction

Of the 37 FGM patients 18 women returned the survey (response rate 48.6%). A change in treatment satisfaction could be recorded by means of various items of the DTSQc. (see Table 2) Range of satisfaction for each item could be rated between − 3 (least increase in satisfaction) and + 3 (greatest increase regarding satisfaction). Patients reported a relevant increase in convenience (mean 2.61) and flexibility (mean 2.56) in the FGM group. A high likelihood of recommending FGM to other GDM patients was reported with a mean of 2.22, as was a higher likelihood of using FGM for future GDM pregnancies with a mean of 2.22. An increase in treatment satisfaction (mean 1.88) and understanding of the disease GDM (mean 1.5) was noted. Changes of perception of hyperglycemia (mean 0.22) or hypoglycemia (mean 0.67) did not change.

**Table 2:** Change of Diabetes Treatment Satisfaction Questionnaire (DTSQc) of FGM pregnancies (*n* = 18): Range of each item of the DTSQc is − 3 (least increase in satisfaction) to + 3 (greatest increase regarding satisfaction)

Changes of …	Mean	Median (IQR)
…Treatment satisfaction	1.88	2.5 (0.75–3)
…Perceived hyperglycemia	0.22	0 (− 2 to 0)
…Perceived hypoglycemia	0.67	0.5 (0–1.25)
…Convenience	2.61	3 (2–3)
…Flexibility	2.56	3 (2–3)
…Understanding of GDM	1.5	2 (0–3)
…Recommendation to others	2.22	3 (2–3)
…Continue in future GDM pregnancies	2.22	3 (2.75–3)

*FGM* flash glucose monitoring, *GDM* gestational diabetes, and *IQR* interquartile range

### Patients' perspective

Subjectively perceived advantages and disadvantages of FGM use in pregnancy were evaluated from written replies of the 18 women who responded to the survey. All of them wrote comments in the category “free field” with a total of 49 comments of which 33 were positive and 16 negative. Thereby, 12 women indicated more advantages than disadvantages, 5 women indicated equal number of disadvantages and advantages of using FGM and one patient, indicated only one disadvantage but no advantage. In total, an average of 2.7 comments were given, of which an average of 1.8 were positive and 0.9 negative.

**Fig. 1:**
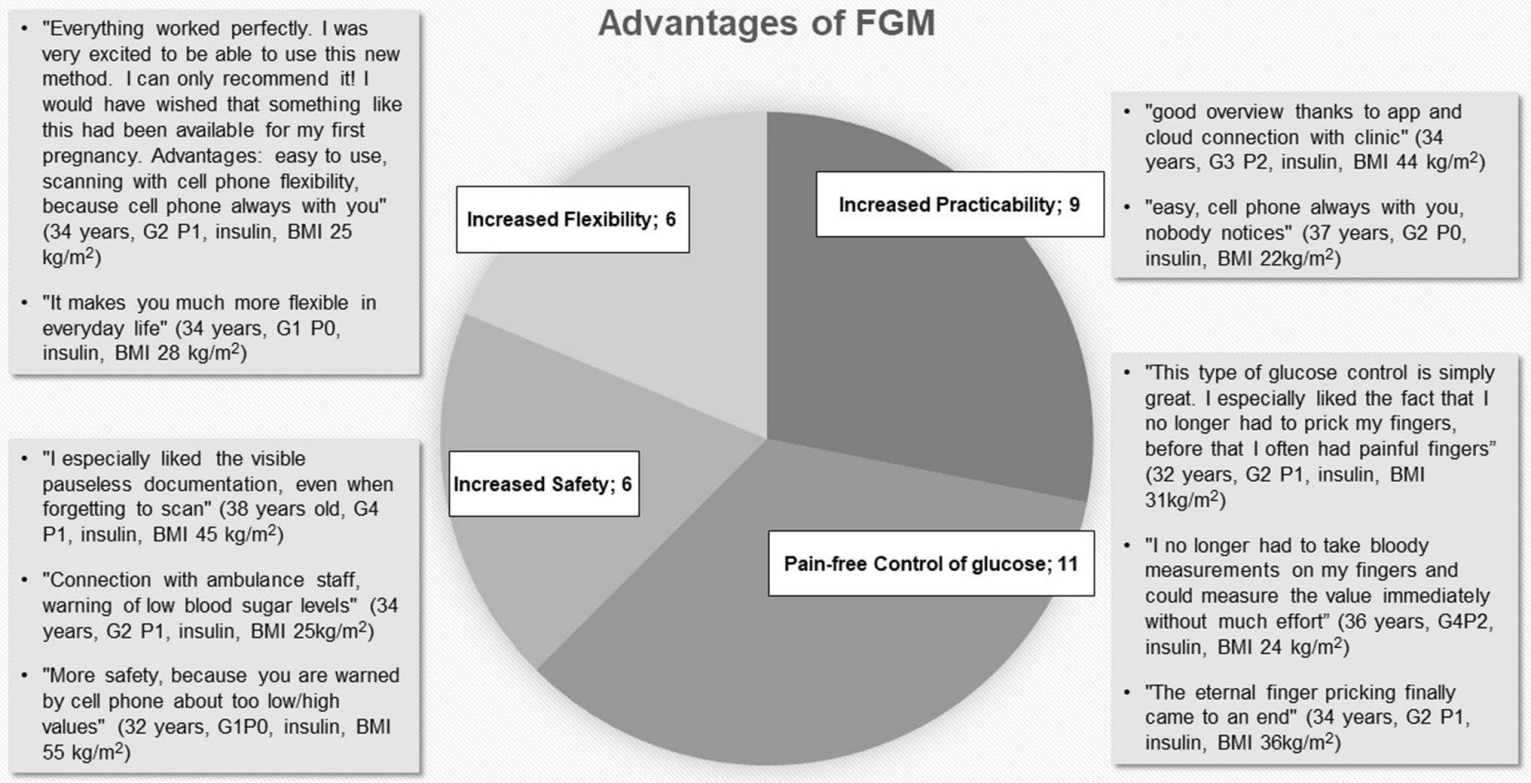
Subjective advantages of FGM use and selected statements from patient interviews

Subjectively perceived advantages identified the following four major topics: increased safety (*n* = 6), pain-free control of glucose levels (*n* = 11), better practicability (*n* = 9) and increased flexibility in daily life (*n* = 6). Selected individual comments about advantages of FGM are shown in Fig. 1. Six patients reported feeling significantly more confident about their health and the health of their child as a result of regularly measuring values, as well as automatic warnings of critical high or low blood glucose levels. 11 women mentioned the change from SMBG to FGM as a significant relief due to more painless control. An increase in practicability and flexibility in everyday life were stated by nine and six women, respectively, confirming that sensor measurement could be better integrated into everyday life, with less effort. Women mentioned especially the convenience of data transfer to their personal phone and integrated reminder functions. With 9 patients stating the improved practicability and 11 the release in pain while controlling their glucose we can state, that the goal of increasing the comfort of our patients during GDM management was achieved.

**Fig. 2:**
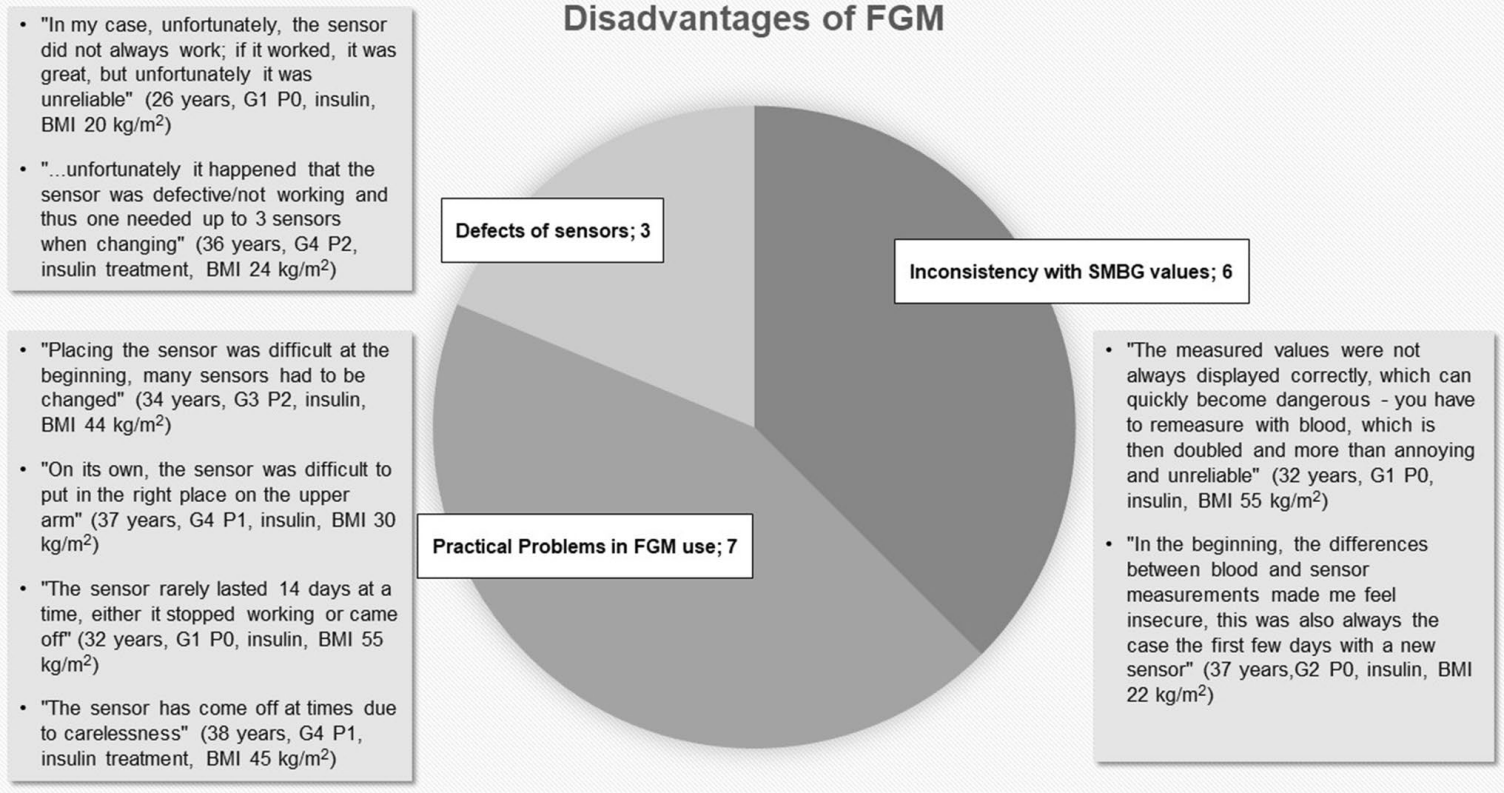
Subjective disadvantages of FGM use and selected statements from patient interviews

Three topics could be identified as possible subjective disadvantages: Varying values (*n* = 6), defect sensors (*n* = 3) and practical problems in use (*n* = 7) and are presented in Fig. 2. Some patients (*n* = 6) complained about the strongly deviating or inaccurate values that occurred in some cases. This caused a feeling of insecurity in some women. In the course of treatment, some patients (*n* = 3) experienced defects of the sensor, sometimes more frequently, which resulted in additional work them. Criticism also related to problems with the application (*n* = 7). Women indicated that there were problems with the proper placement of the sensor. They also reported the sensor detaching or coming off too soon. Cosmetic problems, such as the “visibility of the sensor” (31 years, G1P0, insulin, BMI 29.4 kg/m^2^) and “high acquisition costs” (31 years, G1P0, insulin, BMI 30.9 kg/m^2^) were also mentioned. Criticism was also voiced about the different levels of knowledge of the staff regarding the new therapy at the beginning of this study.

## Discussion

In this study, we were able to demonstrate the safe use of FGM in GDM pregnancies. We could not detect any differences in selected perinatal outcome parameters like rates of C-section, LGA or the need for admission to NICU. High patient’s satisfaction was obtained by the DTSQc questionnaire and the interviews. Given the high prevalence of GDM in approximately 10% in pregnancies, improving the quality of life of these women without compromising quality of care and pregnancy outcomes is of high socioeconomic importance. Although the use of FGM for the care of affected pregnant women has been approved since 2017, to our knowledge, there is a lack of systematically collected data on FGM use and a valid comparison with SMBG in GDM pregnancies that would justify its routine use. Our study provides first evidence of the safety of FGM in GDM pregnancies as well as qualitative data on patient’s treatment satisfaction after changing glucose control from SMBG to FGM.

Perinatal outcome data that are known to be negatively affected by GDM pregnancies were chosen to prove non-inferiority of FGM compared to SMBG. There was no difference in rates of preeclampsia (FGM vs. SMBG: 6.3% vs. 5.5%), preterm delivery (3.3 vs. 2.8%), C-section (26.7% vs. 42.5%), hyperbilirubinemia (35.7% vs. 44.3%), hypoglycemia (6.9% vs. 1.5%) nor NICU admission (3.3% vs. 13.7%). There was a slightly but not significantly higher rate of LGA newborns in the FGM group [20% (*n* = 6) vs. 13.7% (*n* = 10)]. Since women in the FGM group were diagnosed earlier in pregnancy, we hypothesize that these women had a higher metabolic risk due to greater insulin resistance or insulin deficiency, resulting in more LGA newborns, as shown in a study by Liu et al. [30]. Despite the higher rate of LGA neonates and slightly higher hypoglycemia rates in the FGM group, cesarean section rates and NICU admissions were higher in the SMBG group, but without statistical significance.

The subsequent survey on diabetes management and satisfaction after the switch from SMBG to FGM demonstrates possible positive changes, which were noticed. Here, most parameters of the DTSQc showed a significant increase, including flexibility (mean: 2.56) and practicality (mean: 2.61). The increased treatment satisfaction might well be associated with the less complicated measurement method, which was confirmed by patient interviews. The data collected correlates with the results in the study by Kramer et al. [31] in which satisfaction was analyzed after conversion from SMBG to FGM in patients and female patients with type 1 diabetes, they also used the DTSQc for measuring patient’s satisfaction. Similar results were presented in a meta-analysis of the relationship between FGM and patient satisfaction, finding an increase in satisfaction based on the DTSQ [32]. The more frequently perceived hyper- and hypoglycemias in combination with a better understanding of the disease suggest that the patients are more sensitized to hypo- and hyperglycemias and can better classify reactions of the body due to higher insight into the disease. Whether perceived hyper- and hypoglycemia correspond to better training or actual fluctuations that occur cannot be conclusively proven by their study. However, for FGM use to be successful, a certain degree of insight into the disease and an understanding of how to deal with it is essential. In order to make the most of the benefits of FGM, those affected must not only learn to take care of their bodies and recognize dangers, but also receive appropriate training on how the sensor works. In this context, it is also important to train staff appropriately and align the level of knowledge to ensure the best possible care for pregnant women.

However, in order to be able to guarantee satisfactory and perhaps even higher quality care, some points must be analyzed and improved when using the FGM device. The measurement discrepancies noticed by some patients have already been shown in other studies with FGM, but also with CGM use and can be transferred to the measurement accuracy of the sensor in our study [26, 33]. To ensure continued safety and avoid patient concerns, it is necessary to inform patients of any measurement discrepancies that may occur and to discuss with them when an additional blood glucose measurement may be necessary.

This study also has some limitations, which include the small sample size and the monocentric, retrospective study design. In addition, there is likely a bias due to earlier diagnosis of GDM in pregnancies in FGM patients, who are likely to have a more complex metabolic condition that might lead to more adverse outcomes. Nevertheless, a major advantage of this study is the good patient characterization, as well as the unified treatment of both technology groups by a small group of practitioners.

## Conclusion

This sample of GDM patients with FGM use shows the non-inferiority of FGM in comparisons to SMBG glucose control concerning perinatal outcome. FGM use in GDM is safe and well accepted by pregnant women. In addition, it provides increased data on glucose metabolism, further enabling individualized management. The use of FGM for metabolic control should be considered as an equal option in all GDM patients in the future to increase satisfaction and minimize inconveniences due to the diagnosis. In addition to the many benefits, both physicians and patients must be educated about the technical limitations of FGM to ensure safe treatment. We are convinced that FGM can increase the adherence to therapy and well-being of women during their pregnancy. Our study clearly demonstrates that a randomized prospective study evaluating the effect of FGM in GDM pregnancies can be conducted safely.

## Data Availability

The datasets used and/or analyzed during the current study are available from the corresponding author on reasonable request.
